# Selective laser melting of titanium alloy enables osseointegration of porous multi-rooted implants in a rabbit model

**DOI:** 10.1186/s12938-016-0207-9

**Published:** 2016-07-21

**Authors:** Wei Peng, Liangwei Xu, Jia You, Lihua Fang, Qing Zhang

**Affiliations:** Key Laboratory of E&M, Ministry of Education & Zhejiang Province, Zhejiang University of Technology, Hangzhou, Zhe Jiang People’s Republic of China; Department of Stomatology, Zhejiang University of Huzhou Hospital, Huzhou, Zhe Jiang People’s Republic of China

**Keywords:** Implant design, Biomechanics, Titanium (alloys), Multi-rooted implant, Osseointegration

## Abstract

**Background:**

Osseointegration refers to the direct connection between living bone and the surface of a load-bearing artificial implant. Porous implants with well-controlled porosity and pore size can enhance osseointegration. However, until recently implants were produced by machining solid core titanium rods. The aim of this study was to develop a multi-rooted dental implant (MRI) with a connected porous surface structure to facilitate osseointegration.

**Methods:**

MRIs manufactured by selective laser melting (SLM) and commercial implants with resorbable blasting media (RBM)-treated surfaces were inserted into the hind limbs of New Zealand white rabbits. Osseointegration was evaluated periodically over 12 weeks by micro-computerized tomography (CT) scanning, histological analysis, mechanical push-out tests, and torque tests.

**Results:**

Bone volume densities were consistently higher in the MRI group than in the RBM group throughout the study period, ultimately resulting in a peak value of 48.41 % for the MRI group. Histological analysis revealed denser surrounding bone growth in the MRIs; after 4 and 8 weeks, bone tissue had grown into the pore structures and root bifurcation areas, respectively. Biomechanics tests indicated binding of the porous MRIs to the neobone tissues, as push-out forces strengthened from 294.7 to 446.5 N and maximum mean torque forces improved from 81.15 to 289.57 N (MRI), versus 34.79 to 87.8 N in the RBM group.

**Conclusions:**

MRIs manufactured by SLM possess a connected porous surface structure that improves the osteogenic characteristics of the implant surface.

## Background

Dental implants are used routinely in the rehabilitation of partially and completely edentulous patients [1, 2]. However, with the loss of the posterior maxillary and mandibular molars, the use of conventional, standard implants may present a challenge. In fact, some residual alveolar ridges crest in the mesial-distal dimension, resulting in poor support for standard 3.75 or 4 mm diameter implants. In addition, the limitations of a single, wide-diameter implant are apparent in cases of deficiencies in the buccolingual dimension. Bone-grafting procedures are an ideal approach to provide sufficient ridge width for the proper positioning of implants [3, 4]; however, many patients decline this procedure because of the associated time, cost, and morbidity. Additionally, augmentation procedures do not resolve the length of the tissue in the mesial-distal dimension [5].

Evidence suggests that the use of two implants to support a single crown would enable a dentist to rehabilitate the patient without grafting [6–8]. This technique can provide better support against buccolingual and mesiodistal bending. In addition, the use of two implants reduces screw loosening by preventing rotational forces on the prosthetic components [6, 9]. However, current implant systems have limitations with respect to both size and the associated prosthetic component [7]. In many clinical cases, small-diameter implants cannot substitute for standard-sized implants. Narrow-diameter implants for oral reconstruction do not meet the implant occlusal principles; the reduced size of a small diameter implant increases the level of stress on the crestal bone [5].

Additive manufacturing (AM) techniques such as selective laser sintering (SLS) or direct metal laser sintering (DMLS) and selective laser melting (SLM) have benefited the field of biomaterials, especially implant dentistry [10]. AM technologies can be used to directly build three-dimensional (3D) metallic components from metal powders with minimal to no post-processing requirements in combination with a sliced 3D computer-aided design (CAD) model [11]. In combination with cone-beam computed tomography (CBCT) scanning techniques and CAD approaches, custom-made root-analog implants (RAI) for immediate implantation can be fabricated with a biocompatible titanium alloy [12–14]. Laser metal sintering can fabricate functionally graded titanium implants, which are better adapted to the elastic properties of bone [11]. Therefore, SLM-manufactured implants may minimize stress-shielding effects and provide stable long-term fixation.

Porosity and pore size play critical roles in bone ingrowth [15]. Osseointegration is favored by porous implants that improve fixation by creating a mechanical interlock of bone growth into the porous implant structure [16]. The minimum pore size necessary for osseointegration is 100 µm because of bone cell dimensions, migration requirements, and transport; however, pore sizes ≥300 µm are recommended to maximize new bone and capillary formation [17]. By changing the scan speed, powder-feed rates, and the distance between the two metal rods or laser scanners, DMLS can be used to fabricate 3D, interconnected, porous implants [18]. This technology generates porous structures by partially melting the metal powder during the deposition process. This technique is of limited use if the fusion between two particles is not firm, leading to particle detachment. Therefore, the design and direct manufacture of the pore structure by SLM represents a promising alternative.

In this study, an SLM multi-rooted implant (MRI), with a computer-designed surface pore structure, was examined for its potential to overcome the disadvantages of single-rooted implant. The multi-rooted implant with pore structure was evaluated by animal experiment and mechanical tests. A systematic and detailed 12-week study of newbone formation was conducted. Bone contacts around the implants and penetration depth in the porous MRI were evaluated by micro-CT scanning and hard tissue sectioning, respectively. Bonding strength at the bone-implant interface was evaluated by push-out and torque tests, and the value was compared with that resulting from resorbable blasting media (RBM) surface-treated commercial implants.

## Methods

### Preparation of implants

**Fig. 1:**
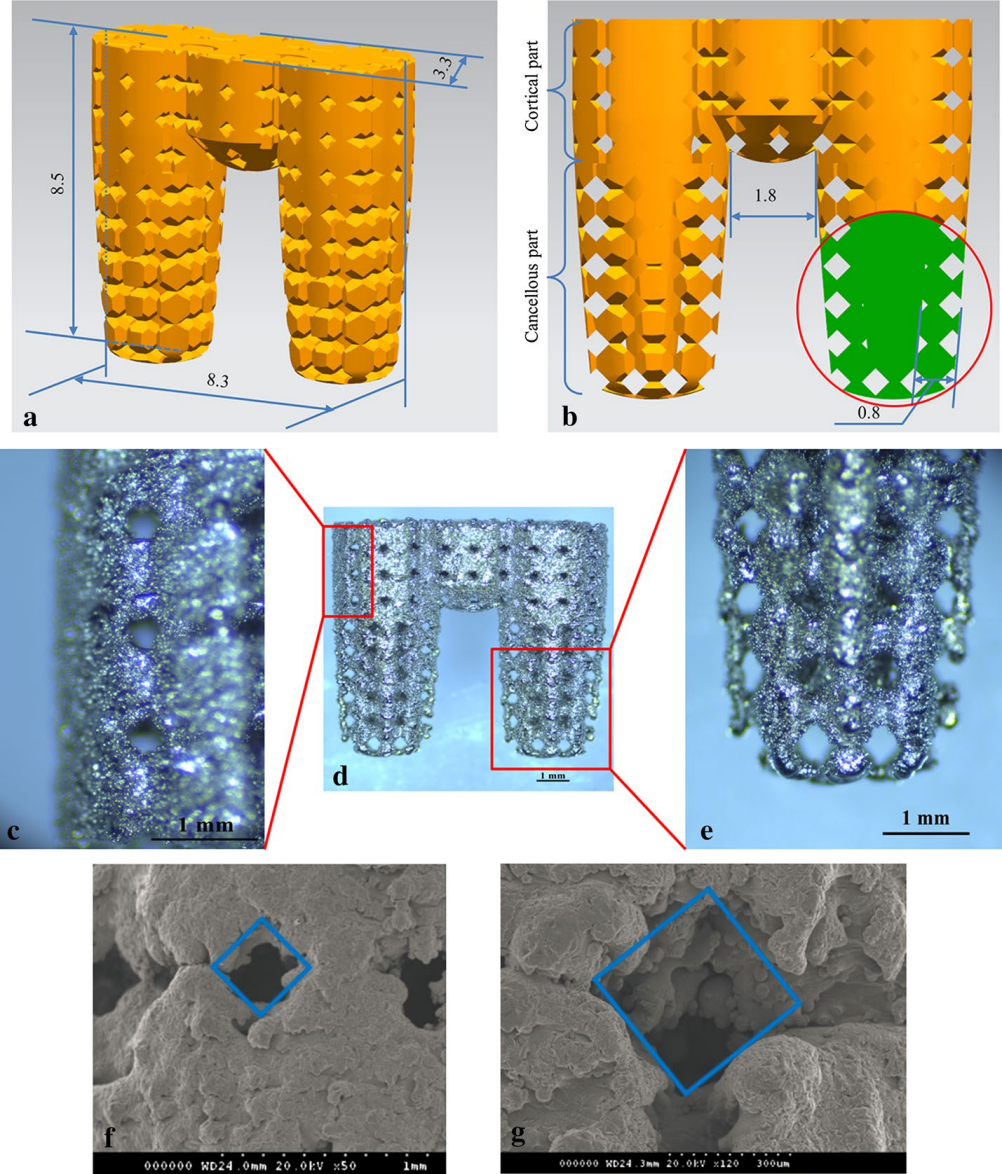
Multi-rooted implant (MRI). **a** Overall implant dimensions. **b** Partial cross-section of the MRI, illustrating the pore structure in detail. **c** The surface of the cortical bone region of fabricated MRI. **d** The overall profile of the fabricated MRI. **e** The surface of the cancellous bone region of the fabricated MRI. **f** Scanning electron microscopy (SEM) image of the cortical bone region of the implant; the pore structure width was approximately 290 µm. **g** SEM image of the cancellous bone region; the pore structure width was approximately 390 µm

A multi-rooted implant CAD model was designed according to the parameters shown in Table 1 and Fig. 1a, b and was manufactured by SLM technology. Samples were made from Ti6Al4 V alloy powder, with a particle size of 15–45 µm. They were processed in an atmosphere of Ar with a powerful Yb fiber laser system (AM250, Renishaw, Gloucestershire, UK) with the capacity to build a volume of up to 250 mm × 250 mm × 300 mm. The diameter of the laser beam spot on the powder surface was 70 µm, with a continuous power of 200 W and a scanning rate of 0.6 m/s. The thickness of the powder layer was 50 µm. To remove residual surface particles, the samples were sandblasted with corundum, and the residual beads inside the micro-pores were cleared by sonication in distilled water (5 min at 25 °C). Following sonication, the samples were immersed in NaOH (20 g/L) and hydrogen peroxide (20 g/L) at 80 °C for 30 min, and further sonicated for 5 min in distilled water [19]. The RBM single-rooted implants were used as control group. The RBM implants had dimensions of Ø 4 mm × 10 mm, with a macroscopic surface area of about 162.7 mm^2^, which is approximately the surface area of the MRIs (the area of RBM implant microstructure and MRI hollow is not considered). All implants were packaged and autoclave-sterilized before surgery. The surface morphology and microstructure of the porous implants were evaluated by scanning electron microscopy (SEM; Hitachi, Tokyo, Japan) and stereomicroscopy (Leica, Wetzlar, Germany).

**Table 1:** Data sheet of multi-rooted implant (MRI)

Region	Porosity %	Pore size (µm)	Thickness (mm)	Length (mm)
Cortical part	26	300	0.8	3
Cancellous part	50	400	0.8	5.5

### Surgical procedure

In this study, 33 adult New Zealand white rabbits of both genders (2.5–3.5 kg) were used to investigate the biocompatibility of the SLM porous MRIs. All animal protocols were reviewed and approved by the Animal Ethics Committee of the Zhejiang University of the Huzhou Hospital. The rabbits were grouped into three experimental time-point groups (4, 8, and 12-weeks) of 11 animals each, including four animals for push-out tests, four for torque tests, and three for histological analysis. Each rabbit had one operation site per tibia and one site per distal femur for a total of four sites. Each rabbit was implanted with two MRIs and two control implants.

The rabbits were anesthetized with injections of sodium pentobarbital (Beijing Chemical Reagent Company, Beijing, China) at a dose of 30 mg/kg body weight in the ear vein. Lidocaine was injected locally into the surgical site before the operation. Before the surgical experiments, the operation areas were shaved, and the skin was sterilized with 2 % povidone-iodine solution and 75 % alcohol. A longitudinal incision was made medially at the distal femur and proximal tibia, and the bone surface was exposed after a blunt dissection through the underlying periosteal connective tissue layer. A surgical guide was used to orient the three sites of the MRI. By intermittent drilling with a low rotary speed and profuse saline (0.9 %, w/v) irrigation, three 3.3-mm holes were prepared. After washing the holes with sterile saline, the test implants were installed into the sites by press fitting. The surgical wound was closed in layers; the periosteum, fascia, and dermal layers were sutured in turn. A Ø 4 mm × 10 mm RBM surface-treated implant was inserted into each of the remaining sites after the implant holes were drilled. The rabbits were allowed to move freely after the operation with no external support and were observed daily for activity. Post-operatively, the animals received 40,000 U penicillin per day for 3 days.

At 4, 8, and 12 weeks post-implantation, 11 animals were euthanized by an overdose of sodium pentobarbital. The bones with the implants were collected and fixed in 10 % neutral buffered formalin.

### Micro-computed tomography analysis

The tibias and femurs of three animals were sectioned into sizes suitable for micro-CT scanning. Before cutting the samples for histological evaluation, the whole bones were scanned by micro-CT (Skyscan 1076, Aartselaar, Belgium) to determine the extent of bone ingrowth. Scanning was performed with a slice thickness of 18 µm, X-ray source at 70 kV, and X-ray intensity at 100 µA. The scanned region was reconstructed with the Skyscan 3D creator “Ant” software. The region of interest (ROI) was selected around the implant and was defined as the area within a 2-mm expanded outline of the implant. The bone volume per total volume (BV/TV,  %) was calculated to represent the portion of mineralized bone tissue.

### Histological evaluation

The bones were cut into 2.0 × 2.0 × 1.0-cm blocks and fixed in 10 % neutral buffered formalin for 5 days. After fixation, the samples were dehydrated in an ascending alcohol series (70, 80, 90, 99, and 100 % ethanol) for 3 days each and embedded in methyl methacrylate resin. Undecalcified ground sections, parallel to the long axis of the implant and the long axis of the tibia/femur, were obtained at a final thickness of 10–15 μm by using a sawing microtome (Leica) at low speed. The sections were stained with toluidine blue. Histomorphometry was performed with a semi-automated digitizing image analyzer system (Nikon, Tokyo, Japan).

### Push-out tests

Push-out tests were conducted with a universal testing machine (Instron, Norwood, MA). A 3.5-mm cylindrical plunger was attached to the crosshead of the test instrument. The bones were supported in a mold with the implant centered over a 5-mm hollow cylinder for the RMB implant and a 10-mm cylinder for the MRI to provide room for the implant to be pushed out with the plunger. The position of the bone in the mold was determined by designing a positioning fixture to ensure that the implant axes aligned with the load-cell. The space between the bone and the mold was filled with die stone for support during the mechanical test. The test was performed at a constant speed of 2 mm/min until the bone-implant interface ruptured. The maximum push-out force (FPmax) was recorded. The FPmax was measured by averaging the results of six tests after removing the highest and lowest of eight push-out test results on different specimens. After the push-out tests, the implant surfaces were observed by SEM (Bruker, Billerica, MA).

### Torque tests

For torque testing, the bones were embedded in a 3D printed mold with a die stone, and a positioning fixture was used to adjust the implant axes orthogonal to the load-cell with a distance of 2 cm. A metal cradle was designed to support the mold to ensure that the center of the plunger aligned with the bottom-clamping device. The test was performed at a constant speed of 2 mm/min, until the bone-implant interface was destroyed. The maximum lateral force (FTmax) was recorded, and the maximum torque (Tmax) was calculated by multiplying the FTmax with the 2-cm distance. The final Tmax was determined by averaging the results of six tests after excluding the highest and lowest results of eight torque tests on different specimens. After torque testing, the implant surfaces were observed by SEM (Bruker, Billerica, MA).

### Statistical analysis

Statistical analysis was performed by using the SPSS v. 19.0 software. Data are reported as median ± standard deviation (SD) at a significance level of *p* < 0.05. One-way analysis of variance (ANOVA) was performed to compare data between experimental periods within the same implant type. The unpaired *t* test was used to compare groups at each time point.

## Results

### Characterization of the MRI

Figure 1c–e shows the manufactured MRI, as observed by stereomicroscopy. The cortical and cancellous bone regions of the MRI had different pore sizes. Processing quality was influenced by the build direction and the fabricated overhanging structure of the pores. In the furthest corner of the bottom, collapsed structures and dross formations were observed; however, the shapes of the top corners were more precise. Similarly, the edges of the pores at the sides of the roots (red arrows) were more prone to structural collapse than the designed model. The pore sizes were observed by SEM (Fig. 1f, g). The surface width dimension was ~290 µm in the cortical area and ~390 µm in the cancellous area.

### Results of micro-CT evaluation

**Fig. 2:**
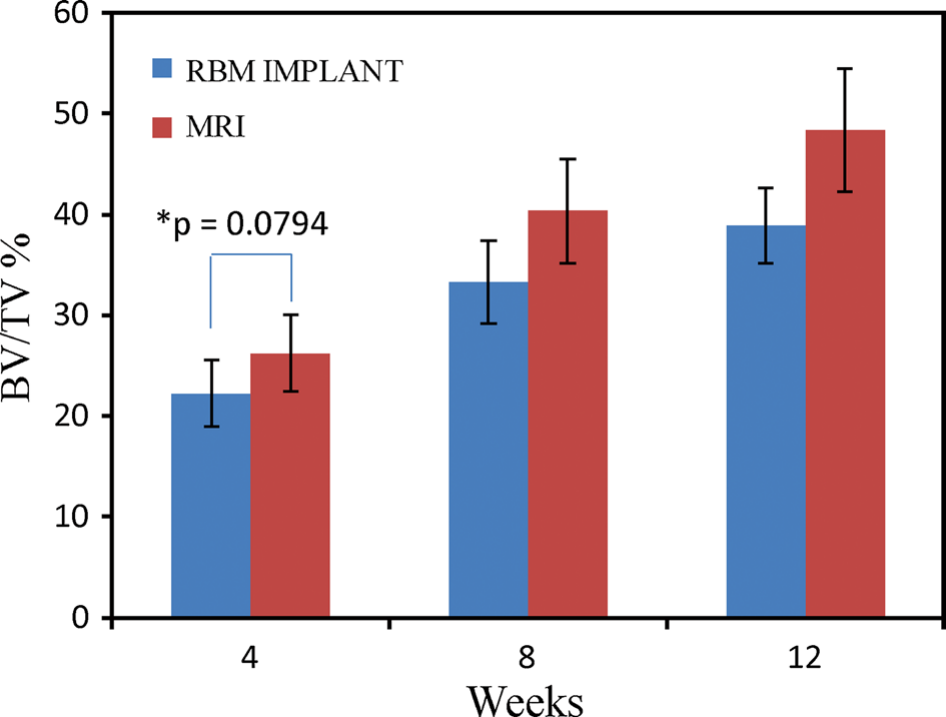
Bone volume per total volume (BV/TV) values of the MRIs and resorbable blasting media (RBM) implants after 4, 8, and 12 weeks. A repeated measures analysis with analysis of variance (ANOVA) and Bonferroni post hoc test showed significant differences (*p* < 0.05) in all cases, except between 8 and 12 weeks within the RBM implant group (*p* = 0.0583); (n = 6, ±SD). *No significance at 95 % (*t*-test)

Micro-CT is a useful technique to quantify bone regeneration around implants. In this study, 3D imaging after 4, 8, and 12 weeks of attachment revealed multi-rooted implant morphologies with a high ratio of bone volume (BV) to total volume (TV). Extensive micro-CT analysis revealed that the MRIs and RBM implants were in physical contact with the neighboring host bone. As shown in Fig. 2, the BV/TV ratio increased remarkably from 26.25 % at 4 weeks to 48.41 % after 12 weeks of MRI implantation. In contrast, for the RBM control implants, the BV/TV ratio increased from 22.24 to 38.92 % over the same period. These results clearly demonstrate that MRIs facilitate excellent bone regeneration in the implant area.

### Histological evaluation of the implants

**Fig. 3:**
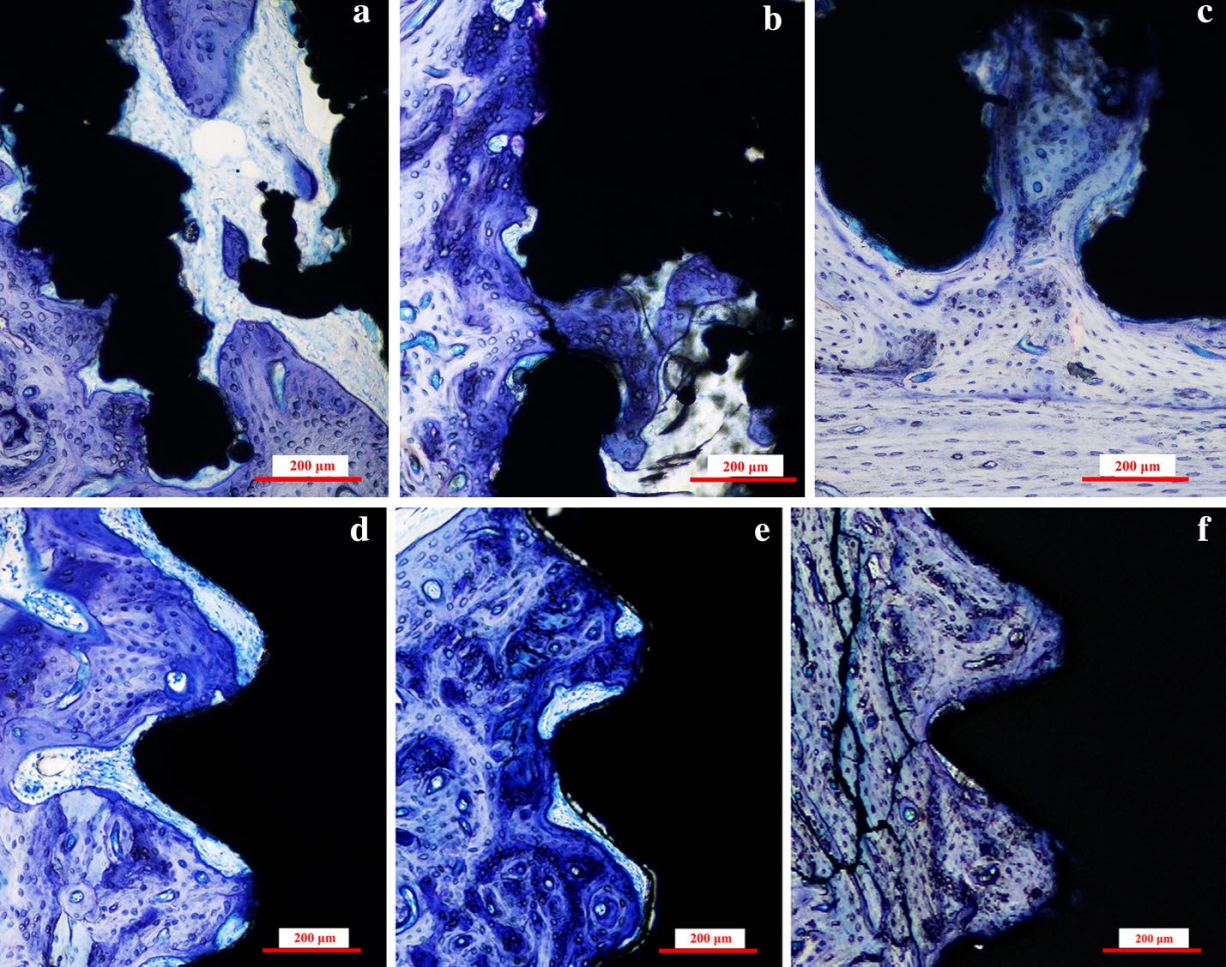
Histological sections of the MRIs and RBM implants. Representative sections of the MRIs in rabbit hind limbs at **a** 4 weeks, **b** 8 weeks, and **c** 12 weeks after implantation, and RBM implants in rabbit hind limbs at **d** 4 weeks, **e** 8 weeks, and **f** 12 weeks after implantation. The sections were stained with toluidine blue

**Fig. 4:**
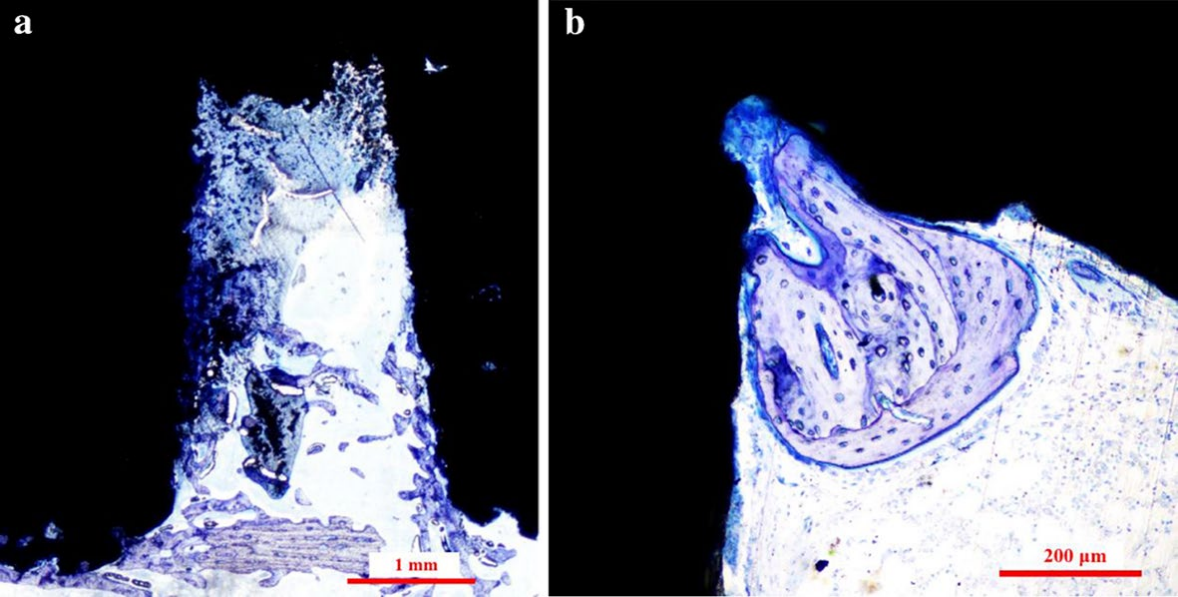
Bone formation at the root furcation area of MRI. **a** A histological section of an MRI, 8 weeks after operation, shows bone growth between the root areas. **b** A representative histological section of an MRI, 12 weeks after implantation, exhibits bone growth at the root furcation

Figure 3 show the histological images of the MRIs and RBM implants at 4, 8, and 12 weeks. No inflammatory reactions or adverse effects were observed at the bone-implant interfaces. During the healing process, bone-forming cells differentiated into osteocytes that were encased in the lacuna within the forming bone matrix. Mature new woven bone with lamellar bone arranged into Haversian systems was observed in the new bone depositions, with osteoid and osteoblasts extending into the implants surface in both implant groups. After 4 weeks of implantation, bone marrow was observed in the pore channels of the MRIs, and the bone tissues grew well on the surface and penetrated into the pores (Fig. 3a). In comparison, the gap between the bone and the RBM implants (Fig. 3d) indicated that the bone in contact with the implant threads was absorbed, likely because of excessive stress. At the end of 12 weeks, the pores of the MRIs were occupied by new bone (Fig. 3c); there was no obvious gap between the bone and the MRI interface. After 8 weeks, considerable bone tissue extended into the space between the two sub roots (Fig. 4a), and after 12 weeks, newly formed bone was observed at the root bifurcation (Fig. 4b).

### Biomechanics results of push-out tests

**Fig. 5:**
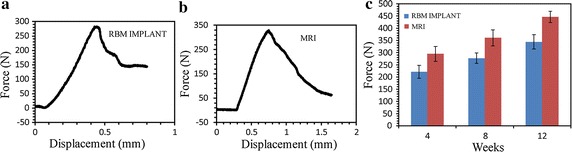
Result of push-out test for the RBM implant and MRI. **a** Representative force–displacement curves for the RBM implant after 8 weeks. **b** Representative force–displacement curves for the MRI after 8 weeks. **c** Maximum push-out forces required for the removal of MRIs and RBM implants. The *graph* plots the average maximum push-out forces of the MRIs and RBM implants after 4, 8, and 12 weeks (n = 6, ±SD). A repeated-measures analysis with ANOVA and Bonferroni post hoc test showed significant differences (*p* < 0.05), except between 4 and 8 weeks in the RBM implant group (*p* = 0.1188) and the MRI group (*p* = 0.1707)

All push-out force–displacement profiles displayed an initial, rapid increase of the load with displacement until a maximum value was reached, corresponding to the de-bonding force (FPmax) between the sample material and the surrounding tissue (Fig. 5a, b). The results of the push-out tests are shown in Fig. 5c. Bonding strength differed between the bone tissue and the implant types. The average maximum push-out force calculated at each time point was consistently higher for the MRI group than for the RBM implant group, and the FPmax difference between the two groups broadened with time.

**Fig. 6:**
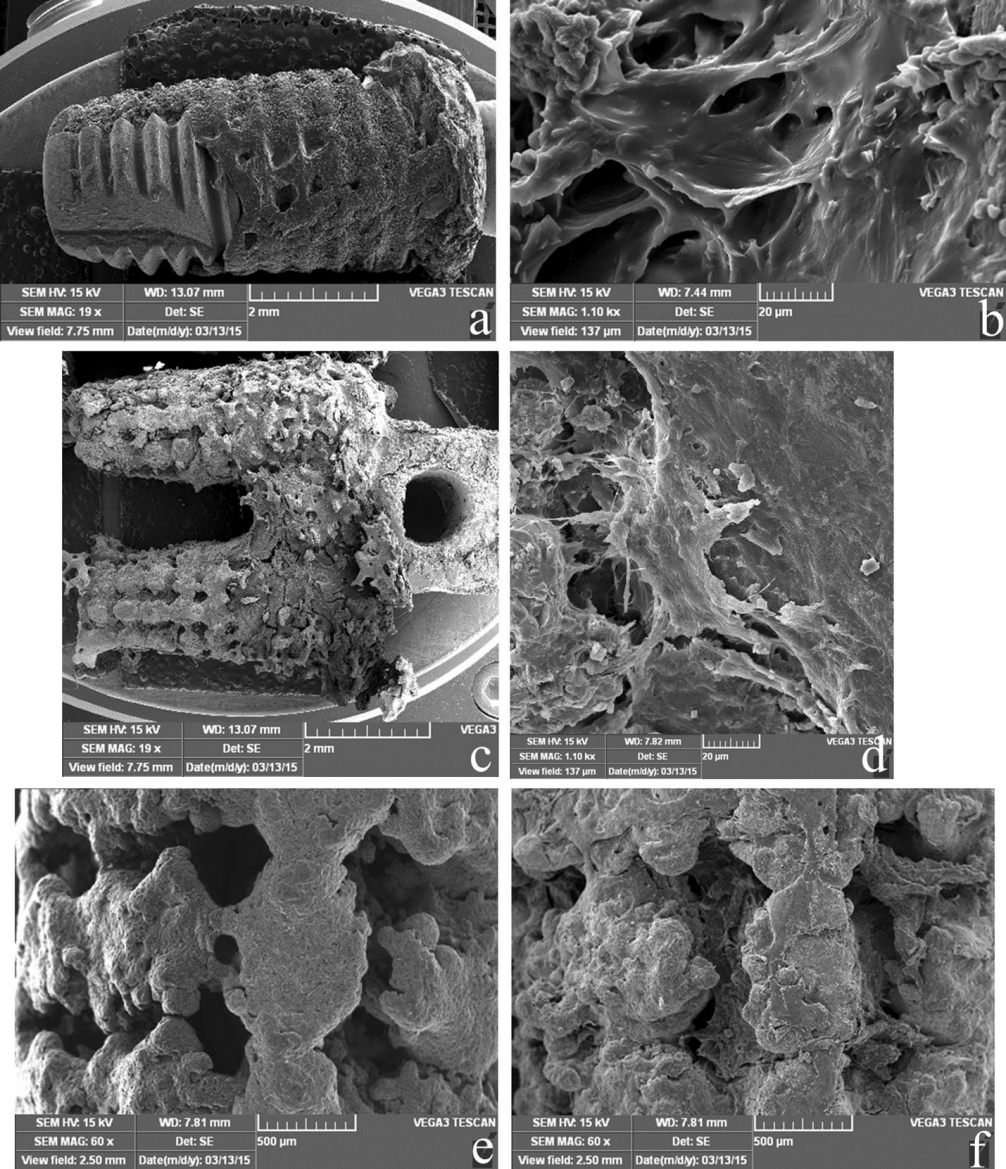
SEM images of the push-out implants 8 weeks after implantation. **a** A global image of an RBM implant. **b** A high-resolution image (×1.1 K) of an RBM implant. **c** A global image of an MRI. **d** A high-resolution image (×1.1 K) of an MRI. **e** An image of the cancellous part of an MRI before implantation. **f** An image of the part at (**e**) 8 weeks after implantation

Figure 6 shows the SEM images of the implant surfaces after the push-out tests, where new bone tissue was deposited in both implant groups. A large number of tissues were observed in the grooved surface of the RBM implant (Fig. 6a); at high magnification, a layer of organic substance with bone matrix covering the implant surfaces was observed (Fig. 6b). Ample attached bone was observed on the MRI surfaces, especially on the cortical part (Fig. 6c). Bone tissues were also observed at the root furcation area. A thick, dense bone matrix layer covered the surfaces, and some tearing due to the push-out tests was noted (Fig. 6d). As shown in Fig. 6f, newly formed bone tissues were clearly observed on the surface and inside the pores of implanted MRIs, in comparison with the pre-implantation implants shown in Fig. 6e.

### Biomechanics results of torque tests

**Fig. 7:**
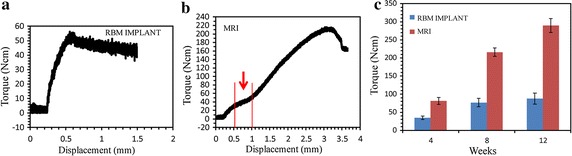
Result of torque test for the RBM implant and MRI. The displacement is measured from the movement of load-cell. **a** Representative torque-displacement curves for the RBM implant after 8 weeks. **b** Representative torque-displacement curves for the MRI after 8 weeks. **c** Maximum torque forces for the MRIs and RBM implants. The *graph* shows the average maximum torque forces of the MRIs and RBM implants over the 4, 8, and 12 week evaluation period (n = 6, ±SD). A repeated measures analysis with ANOVA and Bonferroni post hoc test showed significant differences for all groups (*p* < 0.05), with the exception of 8 and 12 weeks within the RBM implant group (*p* = 0.3463)

Figure 7a, b shows the representative torque-displacement curves of both implant groups. An obvious force plateau appeared only in the MRI curve (indicated by the red arrow in Fig. 7b). In Fig. 7c, the median maximal torque values (Tmax) are shown for each implant at 4, 8, and 12 weeks. The Tmax values increased with time from 4–12 weeks for all implants (Fig. 7c). After the 4-week healing period, the mean Tmax values of the two implant groups were not significantly different. However, at the end of 8 and 12 weeks, the Tmax values tended to be higher in the MRI groups than in the RBM implant groups. The Tmax values of the RBM implant groups improved rapidly between 4 and 8 weeks, but only a slight increase was seen between 8 and 12 weeks.

**Fig. 8:**
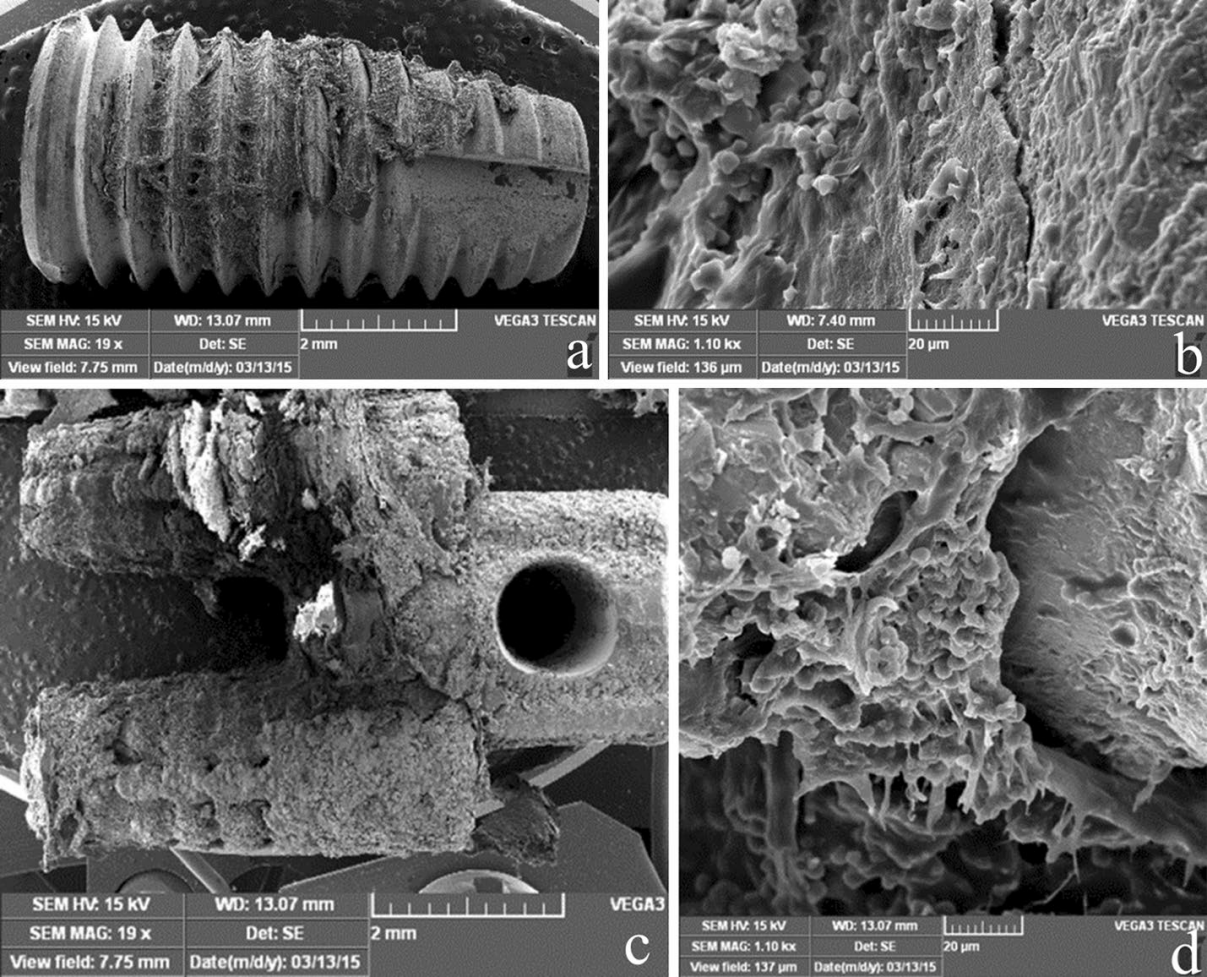
SEM images of the torque test-removed implants 8 weeks after implantation. **a** A global image of an RBM implant, **b** high-resolution image (×1.1 K) of an RBM implant, **c** global image of an MRI, **d** high-resolution image (×1.1 K) of an MRI implant

The SEM images of the RBM implant after torque testing revealed small amounts of bone tissue attached to the implant surface, and the presence of turned-up bone at the periphery of the thread crest surface indicated that the bone tissues had peeled off the implant surface during the test (Fig. 8a). A collagen-free, dense layer that covered and filled the micro-pits, and exhibited cracking, was observed at high magnification in the RBM implants (Fig. 8b). In contrast, the bone tissues grew smoothly on the outer surface and into the channels of the MRIs during the healing process, and bone tissues could be observed at the root furcation area (Fig. 8c). At high magnification, dense bone matrixes were found to have peeled off the surface (Fig. 8d).

## Discussion

Ingrowth of bone tissue into pores is a pre-requisite for successful osseointegration, and it significantly influences the long-term fixation of implants [18]. Pore size, but not pore shape, plays an important role in cell adhesion/migration, vascularization, and new tissue ingrowth [20]. In this study, the pore shape was rhombus, and histological and mechanical results demonstrated that the bone could penetrate into the pore channel. In fact, the pore shape influenced the stress level at the surface, in our previous study, rhombus-shaped pores could reduce pore stiffness [21]. The appropriate pore size for attachment, differentiation, ingrowth of osteoblasts, and vascularization is 200–500 µm [15]. The designed pore size in this study was 300 and 400 µm, while the actual dimensions of the produced pore were ~10 µm smaller (~290 and ~390 µm); however, this decrease in size did not influence the osseointegration results. Our results revealed unequivocally that the newly formed bone tissues could penetrate deeply into the porous MRI (Figs. 3c, 6f).

Biomechanical tests (push-out and torque tests) are used to characterize bonding strength at the bone-implant interface. In this study, push-out tests revealed that the mechanical fixation of the MRIs was stronger than that of the RBM implants. As shown in Fig. 3, newly formed bone tissues penetrated the porous MRI completely, and bound tightly to the MRI trabeculae in the interconnected channels of the implant, thereby generating enough holding and interlocking forces to sustain the implant. At the same time, the tapered profile design of the two sub-roots may increase the friction between the bone and the implant. Therefore, the push-out force was hindered by the coupled bone. In the torque testing process, the primary stress states exerted on both implant groups were different; shear stress was the main load on the RMB implants, while compressive and tensile stresses were the main load types for the MRIs. These different load types led to significantly larger Tmax values for the MRI group compared to the values obtained for the RBM implant group, especially at the 8- and 12-week time points. The results of the torque tests indicated that, compared to the RBM implants, the MRIs exhibited an enhanced ability to resist rotational force. Bone is more resistant to compressive forces than to tensile and shear forces [22]. In the torque-displacement curve for the MRI, a force plateau was observed (Fig. 1b); this plateau is commonly observed in compressive stress–strain curves of cellular materials and is caused by collapse of cells. This plateauing indicates that, during the torque test, the main load on the MRI was compression stress. Together, these results suggest that the porous MRI designed in this study can greatly decrease the shearing stress exerted on the bone during mechanical loads, particularly lateral forces.

The minimum distance needed between adjacent implants has not been determined [23]. For long-term implant success, the existence of a 0.5-mm thick bone around the implant has been advocated, and a lateral biologic width of 1.3 mm around an implant has been suggested [24]. In the present study, the designed distance of the root furcation was 1.8 mm. At the end of 8 and 12 weeks, bone tissue was observed in the space between the roots, as shown by histological sectioning (Fig. 4). SEM images of the MRI after biomechanical tests also revealed abundant newly formed bone attached to the implant at the bifurcation area (Figs. 6c, 8c). These observations indicate that the designed furcation distance of the MRI did not affect the growth of bone tissue.

The most appropriate application of this research in dentistry is in posterior jaw implantation. However, compared to conventional implants, MRIs have several limitations. First the preoperative design plan needs to be optimized, and the orientation of the two roots in the alveolar bone should be considered more carefully. To this end, dentists need to master the planning software, which in turn leads to the problem of how to obtain CT data and increase the cost and preoperative time. Another limitation involves keeping the axis of the two sites parallel during the preparation process. One approach to overcome this limitation is to order surgical guide services, based on a predesigned plan and surgical guide. However, very few companies in China can supply surgical guide services. Therefore, the surgical template is not widely accepted by Chinese dentists. An alternative method is to use ultrasonic tools. However, to enable this, a series of cutter heads that have the same shape as the MRI need be developed.

## Conclusions

A MRI with 3D interconnected pore structural surface and varying porosity was designed and fabricated via SLM. The effects of the MRI on osteoblastic ingrowth, as well as the formation of bone tissues, were investigated systematically. The results indicate that bone can attach to and cover the entire surface of the porous MRI. Histological assessment provided direct evidence that bone tissues penetrated into the channels of the porous MRI after implantation, and that, compared to the RBM implants, the MRIs facilitated fast osseointegration under the same conditions. Biomechanical testing revealed that the porous MRI had a much higher bonding strength at the bone-implant interface than the RBM implant. Future studies will address the long-term stability of the MRI under load after restoration, and develop proper surgical system and tools.

## Abbreviations

AM: additive manufacturing
BV/TV: bone volume per total volume
CAD: computer-aided design
CBCT: cone-beam computed tomography
DMLS: direct metal laser sintering
FPmax: maximum push-out force
FTmax: maximum lateral force
MRI: multi-rooted dental implant
RBM: resorbable blasting media
RAI: root-analog implants
SLM: selective laser melting
SLS: selective laser sintering
Tmax: maximum torque
